# Experimental optical phase measurement approaching the exact Heisenberg limit

**DOI:** 10.1038/s41467-018-06601-7

**Published:** 2018-11-02

**Authors:** Shakib Daryanoosh, Sergei Slussarenko, Dominic W. Berry, Howard M. Wiseman, Geoff J. Pryde

**Affiliations:** 1 Centre for Quantum Dynamics and Centre for Quantum Computation and Communication Technology, Griffith University, Brisbane, Queensland 4111 Australia; 2 Department of Physics and Astronomy, Macquarie University, Sydney, NSW 2113 Australia

## Abstract

The use of quantum resources can provide measurement precision beyond the shot-noise limit (SNL). The task of ab initio optical phase measurement—the estimation of a completely unknown phase—has been experimentally demonstrated with precision beyond the SNL, and even scaling like the ultimate bound, the Heisenberg limit (HL), but with an overhead factor. However, existing approaches have not been able—even in principle—to achieve the best possible precision, saturating the HL exactly. Here we demonstrate a scheme to achieve true HL phase measurement, using a combination of three techniques: entanglement, multiple samplings of the phase shift, and adaptive measurement. Our experimental demonstration of the scheme uses two photonic qubits, one double passed, so that, for a successful coincidence detection, the number of photon-passes is *N* = 3. We achieve a precision that is within 4% of the HL. This scheme can be extended to higher *N* and other physical systems.

## Abstract (web summary)

Measurements of an unknown optical phase have not yet been performed with the ultimate precision, i.e. saturating the Heisenberg limit. Here, Daryanoosh et al. demonstrate this with a precision within 4% of the Heisenberg limit by combining photonic entanglement and multiple passes.

## Introduction

Precise measurement is at the heart of science and technology^1^. An important fundamental concern is how to achieve the best precision in measuring a physical quantity, relative to the resources of the probe system. As physical resources are fundamentally quantised, it is quantum physics that determines the ultimate precision that can be achieved. Correlated quantum resources^2–4^ such as entangled states can provide an enhancement over independent use of quantum systems in measurement.

Quantum-enhanced optical phase estimation promises improvements in all measurement tasks for which interferometry is presently used^5,6^. Such optical quantum metrology can be divided into two distinct tasks. In phase sensing, the goal is to determine small deviations in a phase about an already well-known value—a very specific situation. The use of maximally path-entangled NOON states^7,8^ can, in principle, provide optimal sensitivity for this task^9^. The more challenging task is phase measurement, sometimes called ab initio phase measurement^10^, in which the aim is to determine an unknown phase *ϕ* with no prior information about its value. In this case, the use of multiple passes of the optical phase shift and adaptive quantum measurement^11^, or entanglement and adaptive quantum measurement^12^, have been shown to be capable of surpassing the shot-noise limit (SNL), *V*^SNL^ = 1/*N* (for large *N*). The SNL represents the minimum variance achievable with a definite number *N* of independent samples of the phase shift by a photon. By making correlated samples of the phase shift, these schemes^11–13^ can achieve an asymptotic variance *V* = (*Bπ*/*N*)^2^. This is proportional to, but with a constant overhead *B* > 1 over, the ultimate limit (the Heisenberg limit, HL) of (*π*/*N*)^2^ for the asymptotic ab initio task. To be precise, in terms of Holevo’s variance measure^14,15^, the exact HL for any value of *N* is

1 $$V^{{\mathrm{HL}}} = {\mathrm{tan}}^2\left[ {\pi /\left( {N + 2} \right)} \right].$$

Phase measurement schemes are not limited to optics: equivalent techniques have also used phase shifts of superposition states of single-NV-centre measurements induced by magnetic fields^16,17^, for example.

Here we demonstrate a technique to address this outstanding, fundamental question of quantum metrology: how to measure phase at the exact HL? We show a concrete way to implement the conceptual scheme previously proposed in theory^15^, and implement it experimentally. As in previous photonic ab initio phase estimation experiments, we characterise the quality of our implementation with respect to detected resources—it relies on probabilistic state preparation and measurement schemes, and takes into account only the successful coincidence detections in the calculation of precision. We thus prove the principle of the scheme, which in future can be extended to remove postselective elements.

## Results

### Theory

**Fig. 1:**
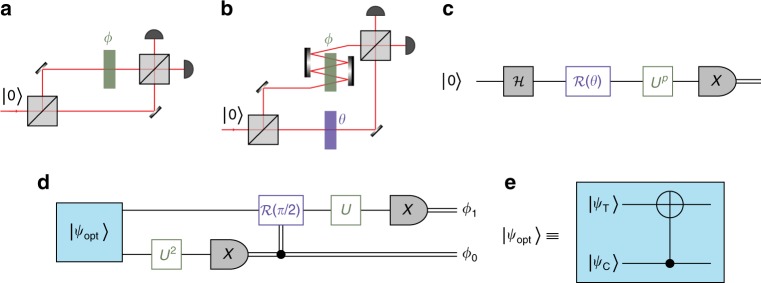
Optical phase measurement concept. **a** Basic interferometric setup for estimation of an unknown phase *ϕ*. **b** Conceptual scheme of an advanced interferometer that includes multiple (*p*) passes of the phase shift *ϕ* and a controllable phase *θ* in the reference arm. **c** Quantum circuit representation of the interferometer shown in **b**. The interferometer is represented by a Hadamard gate ${\cal H}$ and a projective measurement in the *X*-basis, and the application of reference and unknown phases (*p* passes) is represented by unitary operators ${\cal R}(\theta )$ and *U*^*p*^, respectively. **d** Quantum circuit for Heisenberg-limited interferometric phase estimation with *N* = 3 resources. The protocol is extensible to higher *N*, in principle^15^. **e** Quantum circuit for the preparation of the optimal state $|\psi _{{\mathrm{opt}}}\rangle$, Eq. (2), using a CNOT gate with control and target qubits prepared in $|\psi _{\mathrm{C}}\rangle$ and $|\psi _{\mathrm{T}}\rangle$, respectively

We begin by introducing the basic tools and techniques used in this work. The basic concept of optical phase measurement with photons is shown in Fig. 1a. The phase to be measured is inserted in one path of an interferometer; the other path is the reference arm. In the language of quantum information, a photon incident on the first beam splitter (BS) is represented by the logical state |0〉. The action of the BS is modelled by a Hadamard gate ${\cal H}|0\rangle = (|0\rangle + |1\rangle) /\sqrt 2$. The unknown phase shift applied on the path representing |1〉 is implemented by the unitary gate *U*(*ϕ*) = exp(i*ϕ*|1〉〈1|). The last BS prior to detection stages maps the logical *Z*-basis onto the *X*-basis.

A more general protocol may include more sophisticated techniques. The relevant constituents are: the quantum state of the light in the interferometer paths; the possibility of multiple coherent samplings of the phase shift by some photons; and the detection strategy. For example, Fig. 1b generalises the basic single photon interferometer to include *p* ≥ 1 applications of *U*(*ϕ*) and a classically controllable phase, described by ${\cal R}(\theta ) = {\mathrm{exp}}(i\theta |0\rangle \langle 0|)$, on the reference path (representing |0〉). We can also depict this interferometer following the quantum circuit convention, as in Fig. 1c.

For ab initio phase measurement with *N* photons and no multipassing (*p* = 1), it is known theoretically that the HL can be achieved by preparing a path-entangled state^10,18^ and implementing an entangling detection scheme^19^. The problem is that both of these steps are very difficult to do. An alternative way^15^ to achieve the HL uses entanglement across multiple spatio-temporal modes, and multiple applications *p* of the phase gate, combined with the inverse quantum Fourier transform (IQFT) for the measurement. While the IQFT is also an entangling operation, it has been known for some time^20^ that, in this phase estimation algorithm (PEA)^21^, it can be replaced by an adaptive measurement scheme^1^, where individual photons are measured one by one, with the reference phase adjusted after each measurement. This replacement requires the photons in the entangled state to be spread out in time, but suffers no penalty in measurement precision.

Here, we show the practicality of combining entanglement, multipassing and adaptive measurement to achieve the HL. Our Heisenberg-limited interferometric phase estimation algorithm (HPEA)^15^ is illustrated in Fig. 1d. This protocol is based on the standard PEA such that using *K* + 1 qubits yields an estimate *ϕ*_est_ of the true phase *ϕ* with *K* + 1 bits of precision^21^. It involves application of the phase gate *N* = 2^*K*+1^−1 times, with the number of applications being *p* = 2^*K*^, 2^*K*−1^, …, 2^0^ on each successive qubit (photon). Our particular demonstration is an instance of a (*K* + 1=) 2-photon superposition state^15^ that may be used to perform a protocol with *N* = 2^*K*+1^−1 = 3 resources, achieving a variance for ab initio phase estimation of exactly *V*^HL^ (Eq. (1)).

The optimal entangled state for the HPEA is^15^

2 $$\left| {\psi _{{\mathrm{opt}}}} \right\rangle = c_0\left| {{\mathrm{\Phi }}^ + } \right\rangle + c_1\left| {{\mathrm{\Psi }}^ + } \right\rangle ,$$

where

3 $$c_j = \frac{{{\mathrm{sin}}\left[ {(j + 1)\pi /5} \right]}}{{\sqrt {\mathop {\sum}\nolimits_{k = 0}^1 {\mathrm{sin}^2\left[ {(k + 1)\pi /5} \right]} } }},$$

and where $|{\mathrm{\Phi }}^ + \rangle = \left( {|00\rangle + |11\rangle } \right)/\sqrt 2$ and $|{\mathrm{\Psi }}^ + \rangle = \left( {|01\rangle + |10\rangle } \right)/\sqrt 2$ are Bell states. The optimal adaptive measurement^20^ is implemented by measuring the qubits sequentially in the *X-*basis, and, conditioned on the results, adjusting the controllable phase *θ* shifts on subsequent qubits, as shown in Fig. 1d.

### Experimental scheme

**Fig. 2:**
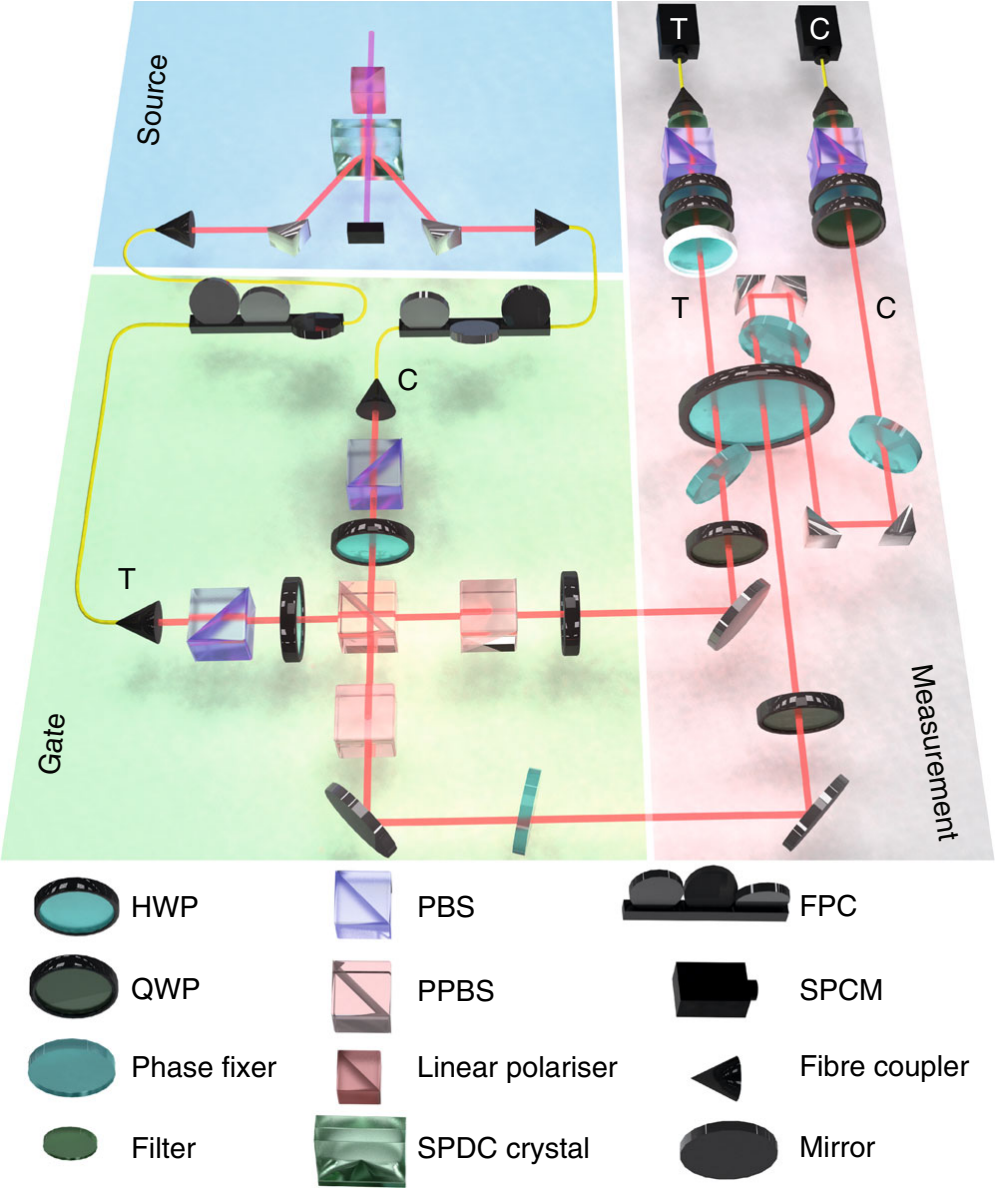
Schematic of the experimental setup. Single photons at 820 nm are generated via a type-I spontaneous parametric downconversion (SPDC) process (blue background) and collected using single-mode fibres and passed into the entangling gate (green background) in order to realise the state $|\psi _{{\mathrm{exp}}}\rangle$. Input polarisation was adjusted with fibre polarisation controllers (FPC). The non-deterministic universal CNOT gate, composed of 3 partially polarising beam splitters (PPBS) and 2 half-waveplates (HWP), performs the state preparation by post-selecting coincidence events between the control and target output ports with success probability 1/9. The area with grey background corresponds to the implementation of the phase estimation. Photons in mode C pass twice through the HWP (acting as a phase shift element), in order to realise the $U(\phi )^2$ operation. Photons in mode T experience the phase shift once (performing the $U(\phi )$ operation). The effect of the feedforward operation, ${\cal R}(\theta )$, is simulated by dialling a HWP (depicted with a white rim), for a fixed time period, with 0 and *π*/8 corresponding to the ON and OFF settings of the control operation. Finally, photons are independently directed to a polarisation analysis unit consisting of a quarter-wave plate (QWP), HWP and a polarising beam splitter (PBS) followed by a 2 nm spectral filter and a single photon counting module (SPCM). See Methods section for further details on the experimental setup operation

**Fig. 3:**
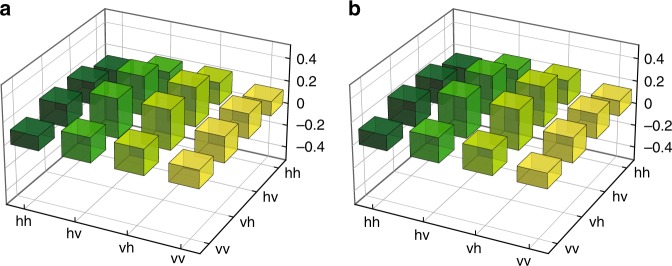
Density matrices of the experimental state and ${\rho }_{{\mathrm{opt}}}$. **a** Real part of the state matrix $\rho _{{\mathrm{exp}}}$ reconstructed with polarisation state tomography. The fidelity of the state with the optimal state $|\psi _{{\mathrm{opt}}}\rangle$, Eq. (2), is $\langle \psi _{{\mathrm{opt}}}|\rho _{{\mathrm{exp}}}{\mathrm{|}}\psi _{{\mathrm{opt}}}\rangle = 0.980 \pm 0.003$, and the purity is ${\mathrm{Tr}}\left[ {\rho _{{\mathrm{exp}}}^2} \right] = 0.965 \pm 0.006$. The density matrix was calculated from ~50,000 twofold coincidence events. Uncertainties in fidelity and purity represent 95% confidence intervals calculated with Monte-Carlo simulation^22^. Imaginary components (not shown) are ≤0.013. **b** Real part of the ideal optimal state $\rho _{{\mathrm{opt}}}$. Note that ${\mathrm{Im}}(\rho _{{\mathrm{opt}}}) = 0$

In our experiment (Fig. 2), we used orthogonal right- and left-circular polarisations instead of paths to form the two arms of the interferometer. We used a non-deterministic CNOT gate, acting on photon polarisation qubits (horizontal |h〉 ≡ |0〉, vertical |v〉 ≡ |1〉), to generate the state in Eq. (2). As shown in Fig. 1e, the control qubit is prepared in the diagonal polarisation state $|\psi _{\mathrm{C}}\rangle = (|{\mathrm{h}}\rangle + |{\mathrm{v}}\rangle )/\sqrt 2$, and the target qubit in the linear polarisation |*ψ*_T_〉 = c_0_|h〉 + c_1_|v〉, so that the output state after the CNOT is the optimal state: $|\psi _{{\mathrm{opt}}}\rangle = \hat U_{{\mathrm{CNOT}}}(|\psi _{\mathrm{C}}\rangle \otimes |\psi _{\mathrm{T}}\rangle )$. Figure 3 shows the density matrices of the experimentally generated state *ρ*_exp_ and the ideal state *ρ*_opt_ ≡ |*ψ*_opt_〉〈*ψ*_opt_|.

The polarisation interferometer, highlighted by the grey background in Fig. 2, used a large half-wave plate (HWP) to implement the unknown phase shift between the arms. Mode C was passed twice through this unknown phase. Another HWP (shown in Fig. 2 with a white rim) was used as the reference phase shift *θ* on mode T, in order to implement the detection scheme.

We implemented the feedforward step non-deterministically, using waveplates that were fixed for each run, combined with postselective sorting of the data based on the results from the detector labeled C. Although this approach would be inadequate for estimation from exactly one shot, it is an accurate way to characterise the performance of the scheme over many repetitions. Table 1 shows how the data were sorted and how phase values were allocated for each shot, according to the detector firing patterns.

**Table 1:** The detection outcome patterns

Outcome in C	*θ*	Successful events in CT	Rejected events in CT
d	0	dd, da	ad, aa
a	*π*/8	ad, aa	dd, da

### Experimental phase estimation

**Fig. 4:**
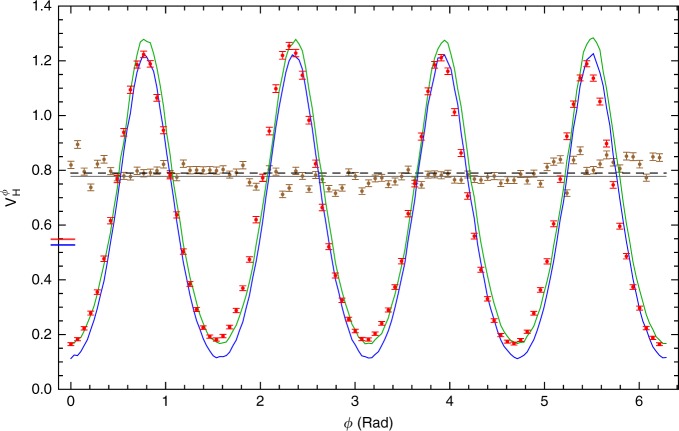
Heisenberg-limited phase estimation with *N* = 3 resources. Red dots represent experimentally measured variance $V_{\mathrm{H}}^\phi$ as a function of *ϕ*. The red horizontal line-segment cutting the left axis shows the optimal protocol Holevo variance $V_{\mathrm{H}} = 0.5497 \pm 0.0007$, determined from these data, while blue line-segment shows the HL. The blue and the green curves represent results of numerical simulations of the variance for the ideal optimal state $\rho _{{\mathrm{opt}}}$ and experimentally prepared state $\rho _{{\mathrm{exp}}}$, respectively. Brown dots represent $V_{\mathrm{H}}^\phi$ for the shot-noise-limited interferometry and the black dashed line represents the measured Holevo variance $V_{\mathrm{H}} = 0.7870 \pm 0.0007$ for the same measurement. The grey solid line shows the SNL. Numerical values for the experimental results and corresponding limits are detailed in Table 2. Each data point was calculated from at least 50,000 twofold coincidence events and the error bars represent 95% confidence intervals calculated with the bootstrap method^23^

**Fig. 5:**
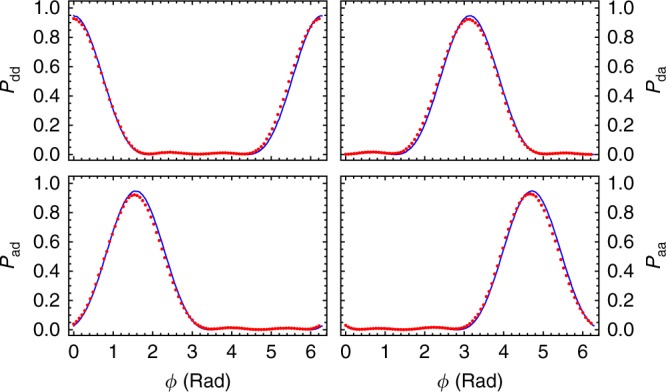
Probability distribution of measurement outcomes. The probabilities of obtaining the four possible $\{ {\mathrm{dd}},\,{\mathrm{ad}},\,{\mathrm{da}},\,{\mathrm{aa}}\}$ measurement outcomes which correspond to four possible $\phi _{{\mathrm{est}}}$ values, for each phase value shown in Fig. 4. The variance $V_{\mathrm{H}}^\phi$ is minimised for those *ϕ* values when one of the probabilities is maximum. Dots are experimental values and lines are numerical simulations that use the experimentally generated $\rho _{{\mathrm{exp}}}$ as input. Error bars, representing the statistical uncertainty due to the finite number of measurement sets, are smaller than the dot size

To characterise the performance of our HPEA, we first calculate the conditional Holevo variance $V_{\mathrm{H}}^\phi$ in the estimates for each applied phase *ϕ* (see Methods section for details on data analysis). Here $V_{\mathrm{H}}^\phi = \left| {\left\langle {\exp [i(\phi - \phi _{{\mathrm{est}}})]} \right\rangle _{\phi _{{\mathrm{est}}}}} \right|^{ - 2} - 1$ for a given *ϕ*, where $\left\langle \ldots \right\rangle _{\phi _{{\mathrm{est}}}}$ indicates averaging over the values of *ϕ*_est_ resulting from the data. Figure 4 shows $V_{\mathrm{H}}^\phi$ for the entire range of *ϕ* ∈ [0, 2*π*). The protocol performs best when *ϕ* = 0, *π*/2, *π*, and 3*π*/2, corresponding to the cases where, to a good approximation, only one of the four possible detection outcomes occur: dd, ad, da, and aa, respectively, as shown in Fig. 5. (Here, d(a) means the diagonal (antidiagonal) polarisation states, which are *X*-basis eigenstates.) It performs worst for intermediate phases. This explains the oscillatory nature of the data in Fig. 4.

As we are interested in evaluating the precision of ab initio phase estimation, we cannot use any knowledge of *ϕ*. Thus we erase any initial phase information by calculating the unconditional Holevo variance $V_{\mathrm{H}} = \left| {\left\langle {\left\langle {\exp [i(\phi - \phi _{{\mathrm{est}}})]} \right\rangle _{\phi _{{\mathrm{est}}}}} \right\rangle _\phi } \right|^{ - 2} - 1$, which averages over *ϕ*. We find *V*_H_ = 0.5497 ± 0.0007, whereas the Heisenberg limit for *N* = 3 resources is *V*^HL^ ≈ 0.5278^24^. As can be seen from the simulation (described in Supplementary Note 1) results in Fig. 4, this 4% discrepancy between the experimental result and theoretical bound can be attributed to the non-unit fidelity of the prepared entangled state with respect to *ρ*_opt_, highlighting the strong correlation between the protocol performance and quality of the prepared state^25^. The small phase offset between the measured data and numerical simulations appears due to a residual phase shift from mirrors and other optical components. This constant phase offset does not influence HPEA precision and can be compensated by a more sophisticated calibration of the setup, or in postprocessing, if required.

For comparison, we perform standard quantum interferometry with three independent photons (see Supplementary Notes 2 and 3 for details). Calculating the Holevo variance for this measurement gives *V*_H_ = 0.7870 ± 0.0007 which is close to the theoretical value of *V*^SLN^ = 0.7778 for the SNL with *N* = 3 resources.

We also compare our results with the theoretically optimal results for other schemes that use a subset of the three protocol components; Table 2. It can readily be observed that our scheme outperforms all those that use two of the components only. While the experimentally measured *V*_H_ is numerically only a little lower than the next best theoretical bound (see Supplementary Note 4 for derivation of theory results), the difference amounts to a 10 standard deviation improvement. We note that arbitrary entanglement can always do the job of multiple passes, by replacing each multipassed photon with a multiple-photon NOON state^7^, split across the two polarisations. Thus our results could, in principle, be reproduced by an entangled state of three photonic qubits, two in one spatio-temporal mode and the third in another, with both modes going through *U*(*ϕ*) once. We rule out such complicated schemes in our comparison by restricting to symmetric entanglement, in which each photon that passes through *U*(*ϕ*) a given number of times is prepared identically. (This is the case for the entanglement in our scheme since each of the two photons passes through *U*(*ϕ*) a different number of times.)

**Table 2:** The Holevo variance for different schemes

Symmetric entanglement	Multipass	Adaptive measurement	*V*_H_
✓	✓	✓	0.5278
✓	✓	✓	0.5497(7) (Exp.)
✓	✗	✓	0.5569^26^
✗	✓	✓	0.5609
✓	✓	✗	0.6547
✗	✗	✗	0.7778
✗	✗	✗	0.7870(7) (Exp.)

## Discussion

We have experimentally demonstrated how to use entanglement, adaptive measurement and multiple passes of the phase shift to perform ab initio phase measurement that outperforms any other scheme, in terms of sensitivity per resource. Our results are very close to the Heisenberg limit for *N* = 3, giving substantial experimental justification to the theoretical prediction that this method can achieve the ultimate measurement sensitivity. While in our analysis we count only photons detected, in twofold coincidences consistent with success of the probabilistic operations, as resources, advances in nascent photon source^27^ and detection^28^ technology, heralded state preparation schemes^29,30^ and deterministic adaptive measurement (with e.g. a Pockels cell) may soon allow saturation of the Heisenberg limit bound even when all the employed resources are taken into account. As quantum phase-sensitive states are susceptible to loss^31^, we expect that similar considerations would apply to the states in our scheme. For small *N*, as we use here, loss has less of an effect on the sensitivity. Future extensions to the scheme will employ *K* + 1 > 2 photons, yielding *N* = 2^*K*+1^−1 resources and a correspondingly decreased phase uncertainty, as quantum logic circuits become increasingly capable of producing large entangled states with high fidelity. We note that while we have implemented this scheme optically, it can be applied to the estimation of any parameter that implements a phase shift between qubit states of some physical system.

## Methods

### Photon source

We used spontaneous parametric downconversion (SPDC) to produce pairs of polarisation-unentangled single photons. Ultrashort pulses from a mode-locked Ti:sapphire laser at 820 nm, with repetition rate of 80 MHz, were upconverted to 410 nm wavelength through a second-harmonic-generation (SHG) process with a 2 mm lithium triborate (LBO) crystal. The SHG beam was collimated with a *f* = 75 mm lens and the IR pump was spatially filtered away with two dispersive prisms. The UV light was focused on a 0.5 mm BiBO crystal to generate photon pairs via a type-I SPDC. The pump power was set to ~100 mW to ensure low probability of double pair emission from the crystal. Using 2 nm narrow band spectral filters, and Excelitas single photon counting modules (SPCMs) with detection efficiency in the range (50–60)%, the overall coincidence efficiency was in the window of (11–13)% with single-detection count rates of ~40,000 counts/s.

### Entangling gate

The single photons produced in the SPDC process were spatially filtered using antireflection (AR) coated single-mode fibres, and sent through the entangling gate to produce a state close to the optimal state *ρ*_opt_. The logical circuit of the gate consisted of three PPBSs, with *η*_v_ = 1/3 and *η*_h_ = 1 for the transmissivity of vertically and horizontally polarised light respectively, to produce a non-deterministic controlled-Z operation^32^. Two of the PPBSs were oriented 90° (around the photon propagation axis) such that *η*_v_ = 1 and *η*_h_ = 1/3, as illustrated in Fig. 2. Two HWPs oriented at 22.5° with respect to the optical axis were used to perform the Hadamard operations required for the correct operation of the CNOT gate. The successful operation of the gate is heralded by the presence of one photon in each output mode of the gate, with overall success probability of 1/9. At the core of this realisation is the non-classical interference that occurs between vertically polarised photons in modes C and T impinging on the central PPBS, Fig. 2. The maximum interference visibility that can be observed with *η*_v_ = 1/3 transmissivity is 0.8. We observed 0.790 ± 0.005 visibility (Supplementary Fig. 1) Hong–Ou–Mandel interference^33^, indicating excellent performance of the gate. In the measurement with three uncorrelated resources, input photon polarisations were set to |h〉, so the photons propagated through the gate without undergoing non-classical interference, but still suffering 2/3 loss in each mode. Photons in mode C were sent to a SPCM and acted as heralds for photons in mode *T*, which in turn were used to perform the shot-noise-limited interferometry.

### Phase shifts and probabilistic adaptive measurements

To encode both unknown and classically controllable phases we proceeded as follows. The prepared state at the end of the entangling gate is ideally in the form of |*ψ*_opt_〉 = c_0_|Φ^+^〉 + c_1_|Ψ^+^〉, Eq. (2), which is a superposition of the Bell states, $|\Phi ^ + \rangle = (|{\mathrm{hh}}\rangle + |{\mathrm{vv}}\rangle )/\sqrt 2$, and $|{\mathrm{\Psi }}^ + \rangle = (|{\mathrm{hv}}\rangle + |{\mathrm{vh}}\rangle )/\sqrt 2$. Here h and v are horizontal and vertical, respectively, polarisation states of a single photon, and encode the logical |0〉 and |1〉 states of a qubit. The linear polarisations were transformed to circular ones prior to the application of the phase shift. This was done by a QWP set at *π*/4, yielding

4 $$\left( {\begin{array}{*{20}{c}} {|{\mathrm{h}}\rangle } \\ {|{\mathrm{v}}\rangle } \end{array}} \right)\mathop{\longrightarrow}\limits^{{U_{\mathrm{Q}}^{(\pi /4)}}}\left( {\begin{array}{*{20}{c}} {e^{i\pi /4}|{\mathrm{r}}\rangle } \\ {e^{ - i\pi /4}|{\mathrm{l}}\rangle } \end{array}} \right).$$

Here $U_{\mathrm{Q}}^{(\gamma )}$ is the unitary operation for a QWP with optic axis oriented at *γ* with respect to horizontal axis. The phase shift of *ϕ* between the right (r) and left (l) circular polarisations could then be applied by setting the 2-inch HWP in Fig. 2 at *ϕ*/4 + *π*/8, producing the transformation

5 $$\left( {\begin{array}{*{20}{c}} {e^{i\pi /4}|{\mathrm{r}}\rangle } \\ {e^{ - i\pi /4}|{\mathrm{l}}\rangle } \end{array}} \right)\mathop{\longrightarrow}\limits^{{U_{\mathrm{H}}^{( - \phi /4 + \pi /8)}}}\left( {\begin{array}{*{20}{c}} {e^{i\phi }|{\mathrm{l}}\rangle } \\ {|{\mathrm{r}}\rangle } \end{array}} \right),$$

where we have ignored the global phase factor, and $U_{\mathrm{H}}^{(\gamma )}$ is the operator of a HWP with optic axis set at *γ*. We implemented the feedforward operation through the same procedure. By analogy with (4) and (5), implementing the feedforward operation by itself, setting the corresponding HWP at *θ*/4 + *π*/8, gives

6 $$\left( {\begin{array}{*{20}{c}} {|{\mathrm{h}}\rangle } \\ {|{\mathrm{v}}\rangle } \end{array}} \right)\mathop{\longrightarrow}\limits^{{U_{\mathrm{Q}}^{(\pi /4)}}}\left( {\begin{array}{*{20}{c}} {e^{i\pi /4}|{\mathrm{r}}\rangle } \\ {e^{ - i\pi /4}|{\mathrm{l}}\rangle } \end{array}} \right)\mathop{\longrightarrow}\limits^{{U_{\mathrm{H}}^{(\theta /4 + \pi /8)}}}\left( {\begin{array}{*{20}{c}} {|{\mathrm{l}}\rangle } \\ {e^{i\theta }|{\mathrm{r}}\rangle } \end{array}} \right).$$

Combining both allowed us to encode the phase shift $\phi - \theta$ between the two arms of the interferometer.

The next step was to perform the adaptive measurements, which we implemented in a probabilistic manner. As the feedback-controlled unitary operation ${\cal R}(\theta )$ has only two settings in this scheme, we set the corresponding HWP at $\theta = 0$ and collected data for a fixed period of time. We recorded only those coincidence events where detector C (Fig. 2) registered a d-polarised photon, as shown in Table 1. We repeated this for $\theta = \pi /8$ and detection of a polarisation at detector C. In other words, when the photon in mode C is projected onto |d〉 (|a〉) state, it is expected that the feedforward unit is in an OFF (ON) setting, equivalent to dialling $\theta = 0{\kern 1pt} (\theta = \pi /8)$ for the HWP acting on the photon in mode T. This provides for characterisation of the protocol performance without active switching.

Each single shot detection (recorded coincidence) provides $\phi _{{\mathrm{est}}} = \pi (\phi _0 \times 2^0 + \phi _1 \times 2^1)/2$. Here, $\phi _0\phi _1 \in \{ 00,\,01,\,10,\,11\} \leftrightarrow \{ {\mathrm{dd}},\,{\mathrm{ad}},\,{\mathrm{da}},\,{\mathrm{aa}}\}$. The probability of obtaining the $\phi _0\phi _1$ result is equal to the number of times $n_{\phi _0\phi _1}$ that this measurement result occurs, divided by the size of the ensemble *n*_ens_ over which the Holevo variance is calculated. Thus from the measurement record we evaluated the true phase *ϕ* using the relation

7 $$\phi \approx {\mathrm{arg}}\left[ {\frac{1}{{n_{{\mathrm{ens}}}}}\mathop {\sum}\limits_{\phi _0 = 0}^1 {\mathop {\sum}\limits_{\phi _1 = 0}^1 {n_{\phi _0\phi _1}} } \,{\mathrm{exp}}\left( {i\phi _{{\mathrm{est}}}} \right)} \right],$$

which becomes exact when $n_{{\mathrm{ens}}} \to \infty$. The conditional Holevo variance $V_{\mathrm{H}}^\phi$ is then calculated according to $V_{\mathrm{H}}^\phi = \left| {\left\langle {\cal S} \right\rangle _{\phi _{{\mathrm{est}}}}} \right|^{ - 2} - 1$, with ${\cal S} = {\mathrm{exp}}[i(\phi - \phi _{{\mathrm{est}}})]$. Finally, the unconditional Holevo variance^18,24^ is calculated as $V_{\mathrm{H}} = \left| {\left\langle {\cal S} \right\rangle _{\phi _{{\mathrm{est}}},\phi }} \right|^{ - 2} - 1$, or, equivalently,

$$V_{\mathrm{H}} = \left| {\left\langle {(V_{\mathrm{H}}^\phi + 1)^{ - 1/2}} \right\rangle _\phi } \right|^{ - 2} - 1.$$

## Electronic supplementary material

Supplementary Information

## Data Availability

The data sets generated during the current study are available from the corresponding authors on reasonable request.

## References

[1] Wiseman, H. M. & Milburn, G. J. Quantum measurement and control. (Cambridge University Press, Cambridge 2010).

[2] Giovannetti V, Lloyd S, Maccone L (2006). Quantum metrology. Phys. Rev. Lett..

[3] Moreau PA (2017). Demonstrating an absolute quantum advantage in direct absorption measurement. Sci. Rep..

[4] Sabines-Chesterking J (2017). Sub-shot-noise transmission measurement enabled by active feed-forward of heralded single photons. Phys. Rev. Appl..

[5] Caves CM (1981). Quantum-mechanical noise in an interferometer. Phys. Rev. D.

[6] Giovannetti V, Lloyd S, Maccone L (2011). Advances in quantum metrology. Nat. Photon..

[7] Dowling JP (2008). Quantum optical metrology—the lowdown on high-N00N states. Quant. Phys..

[8] Nagata T, Okamoto R, O’Brien JL, Sasaki K, Takeuchi S (2007). Beating the standard quantum limit with four-entangled photons. Science.

[9] Slussarenko S (2017). Unconditional violation of the shot noise limit in photonic quantum metrology. Nat. Photon..

[10] Berry DW, Wiseman HM (2000). Optimal states and almost optimal adaptive measurements for quantum interferometry. Phys. Rev. Lett..

[11] Higgins BL, Berry DW, Bartlett SD, Wiseman HM, Pryde GJ (2007). Entanglement-free Heisenberg-limited phase estimation. Nature.

[12] Xiang GY, Higgins BL, Berry DW, Wiseman HM, Pryde GJ (2011). Entanglement-enhanced measurement of a completely unknown optical phase. Nat. Photon..

[13] Berni AA (2015). Ab initio quantum-enhanced optical phase estimation using real-time feedback control. Nat. Photon..

[14] Holevo AS (1984). Covariant measurements and imprimitivity systems. Lect. Notes Math..

[15] Wiseman HM, Berry DW, Bartlett SD, Higgins BL, Pryde GJ (2009). Adaptive measurements in the optical quantum information laboratory. IEEE J. Sel. Top. Quantum Electron..

[16] Waldherr G (2012). High-dynamic-range magnetometry with a single nuclear spin in diamond. Nat. Nanotech..

[17] Nusran NM, Ummal Momeen M, Gurudev Dutt MV (2012). High-dynamic-range magnetometry with a single electronic spin in diamond. Nat. Nanotech..

[18] Berry DW, Wiseman HM, Breslin JK (2001). Optimal input states and feedback for interferometric phase estimation. Phys. Rev. A.

[19] Sanders BC, Milburn GJ (1995). Optimal quantum measurements for phase estimation. Phys. Rev. Lett..

[20] Griffiths RB, Niu CS (1996). Semiclassical fourier transform for quantum computation. Phys. Rev. Lett..

[21] Nielsen, M. A. & Chuang, I. L. Quantum computation and quantum information. (Cambridge University Press, Cambridge 2001).

[22] White AG (2007). Measuring two-qubit gates. J. Opt. Soc. Am. B.

[23] Davidson, A. C. & Hinkley, D. V. Bootstrap methods and their application. (Cambridge University Press, Cambridge 1998).

[24] Berry DW (2009). How to perform the most accurate possible phase measurements. Phys. Rev. A.

[25] Modi, K., Céleri, L. C., Thompson, J. & Gu, M. Fragile states are better for quantum metrology. arXiv:1608.01443 (2016).

[26] Berry, D. W. Adaptive phase measurements, PhD Thesis, The University of Queensland, arXiv:quant-ph/0202136 (2001).

[27] Weston MM (2016). Efficient and pure femtosecond-pulse-length source of polarization-entangled photons. Opt. Express.

[28] Marsili F (2013). Detecting single infrared photons with 93% system efficiency. Nat. Photon..

[29] Barz S, Cronenberg G, Zeilinger A, Walther P (2010). Heralded generation of entangled photon pairs. Nat. Photon..

[30] Ulanov AE, Fedorov IA, Sychev D, Grangier P, Lvovsky AI (2016). Loss-tolerant state engineering for quantum-enhanced metrology via the reverse Hong-Ou-Mandel effect. Nat. Commun..

[31] Knysh S, Smelyanskiy VN, Durkin GA (2011). Scaling laws for precision in quantum interferometry and the bifurcation landscape of the optimal state. Phys. Rev. A.

[32] Ralph TC, Pryde GJ (2009). Optical quantum computation. Prog. Opt..

[33] Hong CK, Ou ZY, Mandel L (1987). Measurement of subpicosecond time intervals between two photons by interference. Phys. Rev. Lett..

