# Supplementary material for: Experimental optical phase measurement approaching the exact Heisenberg limit
Source: Nat Commun. 2018 Nov 2;9:4606. doi: 10.1038/s41467-018-06601-7 (PMC6214903; doi:10.1038/s41467-018-06601-7)
Supplement: Supplementary file 1 — Supplementary Information [file 41467_2018_6601_MOESM1_ESM.pdf]

Supplementary Information
for
Experimental optical phase measurement approaching the exact Heisenberg limit.

S. Daryanoosh *et al.*

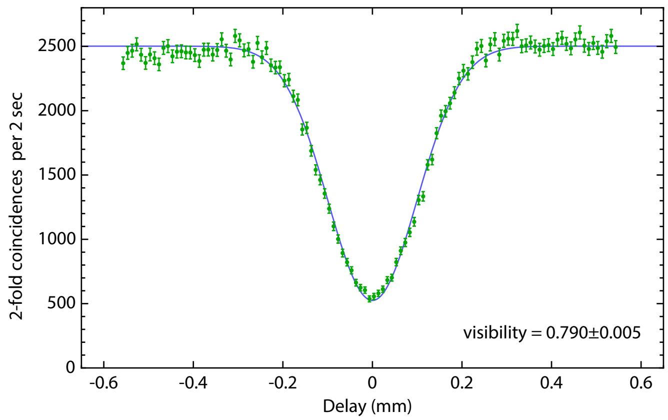

Supplementary Figure 1 | Hong-Ou-Mandel interference at the CNOT gate. Two-photon coincidence rate as a function of the photon indistinguishability, controlled by introducing a propagation delay in one of the photon's path. Maximum theoretical visibility is 0.8.

Supplementary Note 1: Numerical simulation of the HPEA

Consider the Heisenberg-limited interferometric phase estimation algorithm with $K+1 = 2$ (qubits) photons shown in Fig. 1d. Assume the input state is represented by $\hat{\rho}_{\text{in}}$ which allows us to consider mixed input states. The state $\hat{\rho}^{(K)} \in \mathbb{B}_{2^{K+1}}$ ($\mathbb{B}$ denoting Banach space) of the system before the first X-measurement on the K-th photon is

$$\hat{\rho}^{(K)} = \left(\hat{U}^{2^K} \bigotimes_{k=1}^{K} \hat{I}\right) \hat{\rho}_{\text{in}} \left(\hat{U}^{2^K} \bigotimes_{k=1}^{K} \hat{I}\right)^{\dagger}, \tag{1}$$

where

$$U^p = \begin{pmatrix} 1 & 0 \\ 0 & e^{ip\phi} \end{pmatrix}, \qquad I = \begin{pmatrix} 1 & 0 \\ 0 & 1 \end{pmatrix}. \tag{2}$$

Here ϕ is a random unknown phase in the interval $[0, 2\pi)$ to be estimated. To find the outcome of the X-measurement on the K-th photon, we define the following two measurement operators

$$\hat{M}_r^{(K)} = \hat{Q}_r \bigotimes_{k=1}^{K} \hat{I}, \tag{3}$$

where $r \in \{\text{d}, \text{a}\}$ is a measurement result, and $\hat{Q}_r = |r\rangle\langle r|$ is the projection operator onto the X basis of the K-th photon. Thus, the probability of finding the K-th photon in one of the X eigenstates is

$$P_r^{(K)} = \text{Tr}\left[\hat{\rho}^{(K)} \hat{M}_r^{(K)} \hat{M}_r^{(K)\dagger}\right] = \text{Tr}\left[\hat{\rho}^{(K)} \hat{M}_r^{(K)}\right]. \tag{4}$$

Whether the K-th qubit is found in $|\text{d}\rangle$ or $|\text{a}\rangle$ is determined by calling a random number and comparing it with the above probability. Depending on the outcome of this last step, the conditional system state $\hat{\rho}_r^{(K)}$ after the measurement on the K-th photon is obtained according to [1]

$$\hat{\rho}_r^{(K)} = \mathcal{J}\left[\hat{M}_r^{(K)}\right] \hat{\rho}^{(K)} / P_r^{(K)} = \hat{Q}_r \otimes \hat{\rho}_r^{(K-1)}, \tag{5}$$

where $\hat{\rho}_r^{(K-1)} \in \mathbb{B}_{2^K}$ is the reduced state matrix of the other remaining K photons. Here $\mathcal{J}[\hat{O}]\hat{A} \equiv \hat{O}\hat{A}\hat{O}^{\dagger}$ for some arbitrary operators $\hat{O}$ and $\hat{A}$. To proceed with the next step of the protocol, the result of the previous measurement is used to decide whether feedforward should be applied or not. That is, the outcomes $r = \text{a}$ and $r = \text{d}$ correspond to control operation ON and OFF, respectively. Therefore, in the reduced-dimension Hilbert space of the system, the state matrix before the measurement on the $(K-1)$-th photon when the feedback operation is ON can be expressed as

$$\hat{\rho}^{(K-1)} = \hat{\mathcal{U}}^{(K-1)} \hat{\rho}_r^{(K-1)} \hat{\mathcal{U}}^{(K-1)\dagger}, \tag{6}$$

where

$$\hat{\mathcal{U}}^{(K-1)} \equiv \left[\hat{U}^{2^{K-1}}\hat{\mathcal{R}}\left(\frac{\pi}{2}\right)\right]\bigotimes_{k=2}^{K}\hat{\mathcal{R}}\left(\frac{\pi}{2^k}\right), \tag{7}$$

$$\mathcal{R}(\theta) = \begin{pmatrix} e^{i\theta} & 0 \\ 0 & 1 \end{pmatrix}, \tag{8}$$

and if the feedforward is OFF, the state matrix is

$$\hat{\rho}^{(K-1)} = \left(\hat{U}^{2^{K-1}}\bigotimes_{k=1}^{K}\hat{I}\right)\hat{\rho}_r^{(K-1)}\left(\hat{U}^{2^{K-1}}\bigotimes_{k=1}^{K}\hat{I}\right)^{\dagger}. \tag{9}$$

Measurement on the $(K-1)$-th photon is described in the same way as that of the K-th photon. That is, by changing $K \to K-1$ we can use Supplementary Eqns. (3)-(9) to find the measurement result and the reduced state $\hat{\rho}_r^{(K-2)} \in \mathbb{B}_{2^{K-1}}$ of the system. These steps are repeated for each qubit until the 0-th one, for which, the measurement operator is simply the projector $\hat{M}_r^{(0)} = \hat{Q}_r$. Finally the same procedure as described in Methods is employed to calculate the Holevo variance.

Supplementary Note 2: Shot-noise limit analytical calculation

The asymptotic limit of the phase variance can be calculated in an interferometric phase estimation context to obtain the SNL which amounts to $V^{\mathrm{SNL}} \sim 1/N$. This limit is valid when the number N of resources goes to infinity. When N is finite and small, as is the case here in our experiment, this relation does not hold at all. Instead, we were required to analytically calculate what is the SNL for small N's [2].

Consider the interferometer shown in Fig. 1a without multipassing, that is, $p = 1$. Sending single-photon Fock state into one arm of the interferometer, the probability of detecting a photon in either of the output ports in an ideal experimental situation is given by

$$P(u|\phi,\theta) = \frac{1}{2}\left[1 + u\cos(\phi-\theta)\right], \tag{10}$$

where $u \in \{-1, 1\}$ labels the measurement outcome. Assuming that N measurement results are obtained, we can represent them as a vector $\mathbf{u}_N = (u_1, u_2, \cdots, u_N)$ in which each u_j is defined as above. Therefore, the probability for the sequence of measurement results is given by

$$P(\mathbf{u}_N|\phi,\boldsymbol{\theta}) = P(u_1|\phi,\theta_1)\,P(u_2|\phi,\theta_2)\;\cdots\;P(u_N|\phi,\theta_N), \tag{11}$$

where the adjustable phase is varied according to $\theta_j = j\pi/N$. Now recall the Holevo variance in the phase estimate $V_H = \mu^{-2} - 1$ where the sharpness $\mu = \left|\left\langle e^{i\phi}\right\rangle\right|$. Using the conditional probability given in Supplementary Eq. (11), μ can be written as [2, 3]

$$\mu = \frac{1}{2\pi}\sum_{\mathbf{u}_N}\left|\int e^{i\phi}P(\mathbf{u}_N|\phi,\boldsymbol{\theta})\,d\phi\right|. \tag{12}$$

We can then calculate the Holevo variance for small N's by solving this integral. For our experiment in which $N = 3$, we could easily calculate the sharpness and find the exact standard quantum limit to be $V^{\mathrm{SNL}} = 7/9$. However, as the number of resources increases this calculation gets complex and at some point even impossible to solve the integral exactly as the number of possible results goes up exponentially.

Supplementary Note 3: Shot-noise limit experiment

To measure the SNL we have used the same experimental setup. Unentangled single photons were guided through the interferometer such that one of them was used as a probe system and the other one heralded the presence of the former. Instead of passing three photons once through the phase shift element, we sent three single photons sequentially one after the other and adjusted the controllable phase to θ_j, respectively, for $j = 1, 2$, and 3. For each setting there would be two measurement outcomes u_j. This means for a fixed ϕ there are $2^3 = 8$ possible results (two of which, $\phi_1 = \phi_3 = \pm 1$ and $\phi_2 = \mp 1$, are not useful because they yield no information about the unknown phase). Let $n_{u_j}(\phi, \theta_j)$ represents the number of times that a

particular outcome turns up out of an ensemble size $n_{\rm ens} = \sum_{u_j} n_{u_j}(\phi, \theta_j)$. Therefore, the probability of having the outcome $\mathbf{u}_3 = (u_1, u_2, u_3)$ for three independent measurement is

$$P(\mathbf{u}_3|\phi) = \Pi_j \frac{n_{u_j}(\phi, \theta_j)}{n_{\rm ens}}, \tag{13}$$

and the true phase can be calculated using

$$\phi = \arg \sum_{\mathbf{u}_3} \int P(\mathbf{u}_3|\phi)\, e^{i\phi}\, d\phi. \tag{14}$$

On the other hand, $\phi_{\rm est}$ for three measurement outcomes $\mathbf{u}_3$ is

$$\phi_{\rm est}(\mathbf{u}_3) = \arg \int \Pi_j\ P(u_j|\phi, \theta_j)\, e^{i\phi}\, d\phi. \tag{15}$$

For a given ϕ we proceeded as the following to calculate the sharpness

$$\mu(\phi) = \left| \sum_{\mathbf{u}_3}\ P(\mathbf{u}_3|\phi)\ e^{i(\phi - \phi_{\rm est})} \right|. \tag{16}$$

It is easy to work out the Holevo variance by averaging over ϕ in the same way as before.

Supplementary Note 4: Holevo variance for the protocols in Table II

For the case of adaptive measurements and entanglement but no multiple passes, we consider 3 photons in a single spatio-temporal mode so they are indistinguishable. That is, by construction, the photons are identically prepared; the entanglement is symmetric under photon exchange. This single mode could be over an extended time, so photons can be detected separately, and the controlled phase can be adjusted in between detections. As discussed in the main text, if we had instead considered three distinguishable photons in different modes, then having entanglement and adaptive measurements would be the most powerful scheme possible, and would give the same Holevo variance as using symmetric entanglement, multiple passes and adaptive measurements.

There are a total of 3 phases that need to be optimised. Before the first detection, the controllable phase θ has no effect on the results. This is because the system phase is averaged over, and it is only the relative phases that are important. There are two possibilities for the first detection result, and values of the controllable phase θ need to be chosen for each. There are four possibilities for the first two detection results, and again values of the controllable phase θ after those two detections need to be chosen. This gives six phases, but changing the initial value of ϕ by π reverses the significance of the detection results. Because of this symmetry the number of phases that need be considered is reduced by a factor of 2. In addition, the entangled state needs to be optimised over.

Results for the case where the state is optimal for canonical measurements were given in Fig. 11 of Supplementary Ref. [3], where it was found that the phase variance was slightly above that for canonical measurements for 3 photons. That result shows that it is not possible to achieve the HL in that case, though it leaves open the possibility that slightly better performance (but still not at the HL) could be obtained by optimising over the state as well. The result for that case was given in Fig. 6.10 of Supplementary Ref. [4], and the optimisation over the state gives a very slight improvement for $N = 3$. The exact value obtained was 0.5569202271898053.

For the case with adaptive measurements and multiple passes but no entanglement, there are three general possibilities for $N = 3$.

1. One photon with three passes.
2. One photon with two passes and one photons with one pass.
3. Three single photons with a single pass each.

The first is trivial because there is phase ambiguity so the Holevo variance is infinite. The second can be treated using the approach of Sec. IV of Supplementary Ref. [2], where an equivalent two-mode state in a single time mode is considered. There it was found that the ideal canonical measurement gives a variance of $2/N + 1/N^2$ (see Eq. (4.4)). For $N = 3$ this gives $7/9 = 0.777\ldots$ A result for the third possibility was given in Fig. 6.7 of Supplementary Ref. [4], though with a restricted

optimisation of the adaptive measurements, and obtained a Holevo variance of 0.5609756097560981. We have recalculated the variance with full optimisation over θ, and found that the variance is unchanged.

Finally we consider the case of symmetric entanglement and multiple passes, but no adaptive measurements. There are three possibilities again, and again the case with one photon and three passes is trivial. For the others, it is necessary to optimise over the controlled phases θ and the state. The optimisation over the phases is simpler than for the adaptive case, because the phase does not depend on the detection results. It was found that for one photon with two passes and another with a single pass the minimum Holevo variance was 2. The best result was for three entangled photons in a single mode and single passes, in which case the minimum Holevo variance was 0.6546809936433506. We note that if one were to drop the requirement of symmetry on the entangled state, one could obtain a slightly smaller variance of 0.6054864794870138, using an entangled state across three modes.

These calculations were performed in the following way. First, a state of three successive photons in different spatio-temporal modes can be given as

$$|\psi\rangle = \sum_{j,k,l\in\{0,1\}} \psi_{j,k,l}|j,k,l\rangle, \tag{17}$$

where j, k, and l indicate which polarisation each photon is in. This formalism can also be used to treat multiple photons in the same spatio-temporal mode, by using a symmetric state.

Then the operation of measuring a photon as being in one polarisation or the other on the first mode can be represented by

$$\langle a|\psi\rangle = \sum_{j,k,l\in\{0,1\}} \psi_{j,k,l}\langle a|j\rangle|k,l\rangle, \tag{18}$$

where

$$|a\rangle = \frac{1}{\sqrt{2}}(|0\rangle + (-1)^a|1\rangle), \tag{19}$$

for $a \in \{0,1\}$. It is easy to see that

$$\langle a|j\rangle = \frac{1}{\sqrt{2}}(-1)^{aj}. \tag{20}$$

For three measurement results, a, b, and c, the inner product we need is

$$\begin{aligned}\langle a,b,c|\psi\rangle &= \sum_{j,k,l\in\{0,1\}} \psi_{j,k,l}\langle a|j\rangle\langle b|k\rangle\langle c|l\rangle \\ &= \sum_{j,k,l\in\{0,1\}} \psi_{j,k,l}(-1)^{aj+bk+cl}\end{aligned} \tag{21}$$

Similarly to how described in previous sections, the probability of obtaining the measurement result is given by the absolute value squared:

$$P(a,b,c) = |\langle a,b,c|\psi\rangle|^2. \tag{22}$$

Next we explain how to take the phase into consideration. Without loss of generality we can take the first controlled phase θ_0 to be zero (since we average over ϕ). The second controlled phase θ_1 can depend on a, and the third θ_2 can depend on a and b. The change in the state with these controlled phases and the system phase ϕ is

$$|\psi(\phi)\rangle = \sum_{j,k,l\in\{0,1\}} e^{i[j\phi+k(\phi-\theta_1)+l(\phi-\theta_2)]}\psi_{j,k,l}|j,k,l\rangle. \tag{23}$$

This state is convenient to use for calculation, but will not correspond to the physical state at any stage. In reality the first photon would be detected before the phase $\phi-\theta_1$ is applied to the second photon, and so forth. The important quantity is the inner product

$$\langle a,b,c|\psi(\phi)\rangle = \sum_{j,k,l\in\{0,1\}} e^{i[j\phi+k(\phi-\theta_1)+l(\phi-\theta_2)]}(-1)^{aj+bk+cl}\psi_{j,k,l} \tag{24}$$

which enables us to calculate the probability as a function of ϕ

$$P(a,b,c|\phi) = |\langle a,b,c|\psi(\phi)\rangle|^2. \tag{25}$$

Next, we determine the Holevo variance with sharpness μ given by

$$\mu = \frac{1}{2\pi} \sum_{a,b,c\in\{0,1\}} \left| \int e^{i\phi} P(a,b,c|\phi) d\phi \right| . \tag{26}$$

We can calculate the integral as

$$\int e^{i\phi} P(a,b,c|\phi) d\phi = \sum_{j+k+l+1=j'+k'+l'} e^{i[(j-j')\phi+(k-k')(\phi-\theta_1)+(l-l')(\phi-\theta_2)]} (-1)^{a(j-j')+b(k-k')+c(l-l')} \psi_{j,k,l} \psi^*_{j,k,l}. \tag{27}$$

This formula can be used to minimise the phase variance with various types of measurement. If the measurement is allowed to be adaptive, then θ_1 can depend on a and θ_2 can depend on a and b. If it is not adaptive, then θ_1 and θ_2 would need to be chosen independently of the measurement results. Restrictions on the state can also be imposed, for example by requiring it to be separable between the three modes, or by requiring it to be symmetric between the three photons to correspond to three photons in the one mode.

Supplementary References

[1] Wiseman, H. M. & Milburn, G. J. Quantum Measurement and Control. (Cambridge Univ. Press, 2010).
[2] Berry, D. W. *et al.* How to perform the most accurate possible phase measurements. *Phys. Rev. A* **80**, 052114 (2009).
[3] Berry, D. W., Wiseman, H. M. & Breslin, J. K. Optimal input states and feedback for interferometric phase estimation. *Phys. Rev. A* **63**, 053804 (2001).
[4] Berry, D. W. Adaptive Phase Measurements. PhD Thesis, The University of Queensland, 2001; arXiv:quant-ph/0202136.
